# Editorial: Music and Disorders of Consciousness: Emerging Research, Practice and Theory

**DOI:** 10.3389/fpsyg.2016.01273

**Published:** 2016-08-31

**Authors:** Wendy L. Magee, Barbara Tillmann, Fabien Perrin, Caroline Schnakers

**Affiliations:** ^1^Boyer College of Music and Dance, Temple UniversityPhiladelphia, PA, USA; ^2^Centre de Recherche en Neurosciences de LyonLyon, France; ^3^Brain Injury Research Center, Department of Neurosurgery, University of California, Los AngelesLos Angeles, CA, USA

**Keywords:** music, consciousness, brain injury, coma, arousal, therapy, rehabilitation

“No other diagnosis within the field of neurological rehabilitation carries with it such a vast range of clinical, medico-legal, ethical, philosophical, moral and religious implications.”

(Wilson et al., 2005, p. 432)

Despite the body of research on disorders of consciousness (DOC) that has emerged since the “persistent vegetative state” was first named (Jennet and Plum, 1972), the provision of optimal care for this complex population continues to challenge health, medical and science professionals. This is in notwithstanding continual developments in definitions to enhance diagnostic criteria, for example the definition of the minimally conscious state by Giacino et al. (2002) and the term “Unresponsive Wakefulness Syndrome” to replace “vegetative state” proposed by Laureys et al. (2010). Of central concern is the recovery of consciousness (Whyte, 2014), an ambiguous concept that encompasses both wakefulness and awareness (Royal College of Physicians, 2013). Consciousness cannot be directly assessed using verbal reports in this non-communicative population (Seel et al., 2010). The ability to demonstrate consciousness is further compromised in these patients who show minimal motor responses (often due to spasticity), are poorly aroused, and may have significant cognitive and sensory impairments.

The prescient issue has been accurate diagnosis in order to plan appropriate treatment and identify potential in people with DOC. The incidence of misdiagnosis remains unacceptably high (Schnakers et al., 2009; van Erp et al., 2015). For some time, the auditory modality has emerged as potentially sensitive for indicating awareness in people diagnosed as being in vegetative state (Gill-Thwaites and Munday, 2004; Owen et al., 2005, 2006). This insight has prompted greater exploration of auditory stimulation within research and practice, particularly given the suspected incidence of visual impairment in DOC contributing to misdiagnosis (Andrews et al., 1996). More particularly, auditory stimulation with an emotional valence, such as a familiar voice or the patient's own name, has been shown as helpful when trying to understand residual brain processing and when uncovering consciousness in patients with severe brain injury (Bekinschtein et al., 2004; Laureys et al., 2004; Schiff et al., 2005; Perrin et al., 2006; Di et al., 2007). As music has a well-known relationship with emotional processing (Frühholz et al., 2014; Omigie, 2016), such stimulation might be crucial when assessing the recovery of consciousness in this challenging population.

## Music and DOC

Based in early studies from the 1990's, an interest in using music as a medium for diagnosis, exploration and treatment in DOC has rapidly expanded in recent years, and in particular with the growing research community of music and neurosciences interested in the use of music as a tool for promoting sensory, cognitive and motor stimulation of the normal and pathological brain (see Bigand et al., 2015, for recent reviews). Current behavioral explorations with DOC populations have used music to train behaviors (Charland-Verville et al., 2014) and have demonstrated that music can elicit behavioral responses indicative of arousal (O'Kelly et al., 2013). Music listening has a beneficial effect on cognitive processes in people with DOC (Castro et al., 2015) including increased EEG amplitude in alpha and theta bands suggestive of attentional processes (O'Kelly et al., 2013). Case studies suggest music may have diagnostic value to distinguish between vegetative and minimally conscious states, as well as prognostic potential (Okumura et al., 2014). Clinical music protocols for therapeutic intervention have been developed and music-based diagnostic measures have been standardized (Magee and O'Kelly, 2015).

Interest in this topic is growing at an exponential rate, on the understanding that language impairment is likely following profound brain injury and that music is both an alerting and an emotional stimulus (Castro et al., 2015). It is apparent that energies need to be harnessed, coordinating the efforts of diverse disciplines spanning research, science, and clinical practice. This special issue grew from a desire to strengthen the body of evidence for using music in the assessment, diagnosis, and treatment of people with DOC in order to improve research and practice. We aimed to provide a platform for cross disciplinary discussion to encourage multiple perspectives. This international collection helps to consolidate the foundations of this field of study for future work on the topic.

Our call for papers was responded to by scientists, researchers and clinical practitioners, resulting in nine articles. Three papers are Original Research Articles, three are Opinion Papers, two are Reviews, and one is a Perspective paper. The research groups span seven countries (Austria, France, Germany, Italy, Russia, UK, USA) and include 28 authors.

## The neuromusicology of disorders of consciousness

Different methodologies have been used to investigate the effect of music on the brain functioning of DOC patients. The present issue reports four articles in which the interest of autonomic recordings, EEG and functional MRI connectivity are evaluated. First, Kotchoubey et al. reviewed empirical data on different functions (perception, cognition, emotions, and motor functions) that musical stimulation can address in DOC patients. This is supported by an original study in which Riganello et al. investigated the link between music listening and heart rate variability, and in which they found differences between DOC and healthy subjects, characterized by lower values in VS/UWS patients. Lord and Opacka-Juffry, in an opinion paper, claim that EEG studies of coherence can be used to assess the effects of auditory stimuli in DOCs, for example during the course of music therapy. According to them, these studies could help to understand whether changes in connectivity across the mesocircuit pathways are an indicator of the conscious state and neuroplasticity. Cerebral connectivity is also investigated in an original contribution, with functional MRI methodology, in which Heine et al. showed that the auditory network is more connected with the left precentral gyrus and the left dorsolateral prefrontal cortex, and the external network with the temporo-parietal junction, during preferred music listening (as compared to a noise control condition). Thus, this is a first demonstration that music listening might have effects, in DOC patients, on networks implied in rhythm and music perception, as well as in autobiographical memory.

## Clinical applications of music with DOC: Theory and practice

Perspectives on potential clinical applications were discussed in five papers. The theoretical principles for the efficacy of sensory stimulation vs. music stimulation is discussed by Schnakers et al. Further, investigation is warranted as methodological issues, including small sample sizes, agreement on core outcomes, and sensitivity of measures, have all contributed to limiting adequate investigations to draw conclusive results. Nevertheless, the preliminary findings obtained until now encourage further investigations to confirm the benefit of music, not only in the detection of consciousness, but also in the treatment of patients with severe brain injury.

The theoretical position for using music is developed by Perrin et al. who highlight that the selection of stimuli with emotional, autobiographical or self-related characteristics is critical in people with DOC. In their review, they position music as a potential tool for engaging both internal and external networks, facilitating awareness of self in addition to awareness of environment and contributing to long-term cognitive improvement.

Research into pediatric DOC is a neglected topic with no measures currently standardized for use with children and adolescents and no guidelines for clinical practice. Pool and Magee continue the discussion comparing sensory stimulation and music with pediatric DOC, presenting an overview of the theoretical literature. Music is a primary modality for learning and development in normal pediatric populations, due to its utility for cross-modal application, cognitive priming, and emotional stimulation. As the incidence of visual impairment in children and youth with DOC is notably high, the authors argue that music provides a developmentally appropriate medium that is also accessible. Following from this, Magee et al. explore the clinical utility of a music-based measure with a pediatric DOC population. Data from four cases examine outcomes from the music therapy assessment tool for awareness in disorders of consciousness (MATADOC), a protocol and measure that have been standardized for adults. The results suggest higher functioning in the visual and auditory domains when compared to several external reference standards whose protocols do not optimize non-verbal auditory stimuli. This is promising for clinical practice and supports further investigation into using music as a diagnostic and treatment tool for pediatric DOC.

Lastly, Vogl et al. present the case for a neuroanthropological approach in research with DOC patients for obtaining knowledge and influencing practice. This approach positions music as representing meaning from the individual patient's perspective. Two case descriptions illustrate how quantitative PET data can be augmented by qualitative behavioral observations. The cases illustrate how clinical music protocols can be adapted to individual needs, and that these investigations can contribute to the multidisciplinary team's understanding of how to manage the patient's daily life and environment.

## Author contributions

All authors listed, have made substantial, direct and intellectual contribution to the work, and approved it for publication.

### Conflict of interest statement

The authors declare that the research was conducted in the absence of any commercial or financial relationships that could be construed as a potential conflict of interest.
